# Expression of BACE1 in the Rat Carotid Body

**DOI:** 10.3389/fphys.2020.00505

**Published:** 2020-05-20

**Authors:** Chaohong Li, Baosheng Zhao, Ya-nan Fan, Xianglei Jia, Yuzhen Liu

**Affiliations:** ^1^Henan Key Laboratory of Neural Regeneration and Repairment, Henan Neurology Institute, The First Affiliated Hospital of Xinxiang Medical University, Xinxiang, China; ^2^Department of Thoracic Surgery, The First Affiliated Hospital of Xinxiang Medical University, Weihui, China

**Keywords:** carotid body, BACE1, ROS, cyclic intermittent hypoxia, plasticity

## Abstract

This study explored the expression of BACE1 (β-amyloid precursor protein cleaving enzyme 1) in the rat carotid body and the effect of CIH (cyclic intermittent hypoxia) on the expression of BACE1. We found that BACE1 was expressed in the rat carotid body and located in the nerve endings and type II cells but not in type I cells. CIH reduced BACE1 level in the carotid body, and reoxygenation or ROS scavenger alleviated this reduction. Furthermore, we found that CIH augmented the mRNA level of PGC-1α but attenuated the mRNA level of BACE1 in the carotid body. Taken together, our results suggest that CIH promotes the production of ROS that upregulates the level of PGC-1α, which may in turn inhibits the transcription of BACE1, and that a reduction in the BACE1 level may be related to CIH-induced reversible and ROS-dependent carotid body plasticity. Our study provides a new candidate molecule for further study of the mechanism of carotid body plasticity.

## Introduction

BACE1, also called β-amyloid precursor protein cleaving enzyme 1 or β-secretase, is a transmembrane aspartyl protease that has gained significant attention as a crucial protease for the generation of the β-amyloid peptide, a crucial initiator in the pathogenesis of Alzheimer’s disease (AD) (Vassar et al., 1999). BACE1 is expressed not only in the brain but also in the peripheral nervous system. A wide variety of membrane proteins have been identified as potential BACE1 substrates, and some of these substrates have been linked to synaptic function (Filser et al., 2015; Munro et al., 2016) and the peripheral nerve regeneration process (Farah et al., 2011; Tallon and Farah, 2017). The role of BACE1 in the peripheral nervous system is dependent on its substrate and is related to pathophysiological conditions. For example, under certain physiological conditions, BACE1 is required for peripheral nerve myelination and axonal bundling via the cleavage of type III neuregulin 1 (NRG1) (Willem et al., 2006; Hitt et al., 2012). However, after injury to peripheral nerve axons, a decrease in BACE1 level or activity can enhance peripheral nerve axonal regeneration by promoting debris clearance (Farah et al., 2011) or by reducing the cleavage of close homolog of L1 (CHL1) and L1, thus increasing their interactions with Schwann cells (Tallon and Farah, 2017). The transcription of BACE1 is positively or negatively regulated by a number of transcription factors and cofactors. Peroxisome proliferator-activated receptor gamma coactivator 1-alpha (PGC-1α) is a coactivator of peroxisome proliferator-activated receptor γ (PPARγ) and plays a powerful role in reactive oxygen species (ROS) metabolism (St-Pierre et al., 2006). It has been shown that PGC-1α reduces BACE1 levels by repressing transcription in the BACE1 promoter region (Wang et al., 2013) or by enhancing BACE1 degradation (Gong et al., 2013).

The carotid body (CB) is a small cluster of peripheral chemoreceptor located bilaterally near the bifurcation of the common carotid artery, and it consists of two major cell types: type I cell (glomus cell) and type II cell (sustentacular cell). Type I cells, as the oxygen-sensitive cells in the CB, transduce changes in arterial oxygen tension (PO_2_) into afferent nerve signals through the carotid sinus nerve to the nucleus tractus solitarius (NTS). Through the NTS, the changes are relayed to other centers in the brain, resulting in increased respiration and a coordinated cardiovascular response. Type II cells are glial fibrillary acidic protein (GFAP)-positive glia-like cells that envelop clusters of type I cells. It has been demonstrated that cyclic intermittent hypoxia (CIH) results in plasticity of the carotid chemoreceptor response (Kumar and Prabhakar, 2012). For example, CIH exposure for 10 days induces a functional plasticity termed sensory long-term facilitation (sLTF) in the rat CB, and this phenomenon is completely reversed after reoxygenation with room air for 10 days (Peng et al., 2003). Further studies have found that CIH-induced sLTF is associated with the NOX-induced ROS signaling in the CB (Peng et al., 2003, 2009). However, the mechanisms by which ROS is involved in the plasticity of the CB responsible to CIH are uncertain.

We previously found that BACE1 was expressed in rat adrenal medulla (Fan et al., 2020). The CB and the adrenal medulla chromaffin cells originate from the neural crest; they are both peripheral chemoreceptors and are important components of the homeostatic acute O_2_ sensing system (Gao et al., 2019). Whether BACE1 is expressed in the CB and involved in the regulation of CIH-induced CB plasticity is undefined yet. Therefore, we examined the expression of BACE1 by RT-PCR and immunostaining in rat CB. We demonstrated that BACE1 was expressed in type II cells and nerve endings but not in type I cells in the CB. Moreover, we found that CIH reversibly reduced BACE1 protein level in a ROS-dependent manner. The mechanism by which CIH inhibits BACE1 expression might be through upregulation of PGC-1α, which in turn inhibits the transcription of BACE1. Our study provides BACE1 as a new candidate molecule for further study of the mechanism of carotid body plasticity.

## Materials and Methods

### Reagents and Antibodies

RNAlater, the QuantiTect Reverse Transcription Kit and the HotStarTaq^®^ Master Mix Kit were purchased from Qiagen (Valencia, CA, United States). Trizol reagent was purchased from Thermo Fisher Scientific (Waltham, MA, United States). Rabbit anti-BACE1 and mouse anti-neurofilament (NF) antibodies were all purchased from Abcam (Cambridge, MA, United States). Mouse anti-GFAP, Alexa Fluor 488 goat anti-rabbit IgG and Alexa Fluor 555 goat anti-mouse IgG antibodies were purchased from Cell Signaling Technology (Waltham, MA, United States). The GTVisionTM III Detection System/Mo & Rb Kit and the DAB Detection Kit were purchased from Gene Tech (Shanghai, China). Isoflurane, a mouse anti-TH antibody and manganese (III) 5,10,15,20-tetra(4-pyridyl)-21H,23H-porphine chloride tetrakis (methochloride) (MnTMPyP) were ordered from Sigma (St. Louis, MO, United States).

### Animals

Adult male Sprague-Dawley (SD) rats were obtained from Beijing Vital River Laboratory Animal Technology Co., Ltd. (Beijing, China), and the body weight (wt) at the beginning of the procedure was between 220 and 230 g. The rats were housed under room temperature with a 12 hr day/night cycle with chow and water *ad libitum*. All animal experimental protocols were approved by the Institutional Animal Care and Use Committee of The First Affiliated Hospital of Xinxiang Medical University.

### Cyclic Intermittent Hypoxia Exposure, Reoxygenation, and Application of MnTMPyP

The CIH exposure, reoxygenation and control procedures were similar to those described in a previous report (Liu et al., 2009). Briefly, SD rats were moved from their home cages to the special cages placed in the chamber of Oxycycler Model A84XOV (Biospherix, Redfield, NY, United States) and were exposed to CIH or air. For the CIH group, the chamber was flushed with 100% N_2_ until the fraction of inspired oxygen (FiO_2_) reached 10% for 3 min. The FiO_2_ level was then returned to 21% by flushing with 100% O_2_ for the remaining time. The exposure cycle was repeated every 6 min for 8 hr/day from 8:30 to 16:30 for 2 weeks. This paradigm of CIH is a mild paradigm as it has 10 episodes per hour. For the control group, the control rats underwent the same exposure, but the chamber was flushed with compressed air. For the CIH plus reoxygenation (CIH + ReOx) group, the rats were returned to room air for 2 weeks after an initial 2 weeks of CIH exposure. For the antioxidant treatment (CIH + MnTMPyP) group, MnTMPyP at a dose of 5 mg/kg/day was administered via intraperitoneal injection (ip) into the rats each morning before the rats were exposed to CIH. For the vehicle control group (CIH + Vehicle), the CIH rats were administered with saline instead of MnTMPyP via ip each morning before the rats were exposed to CIH. After completion of each protocol, the animals were assigned to the following studies.

### Preparation of Paraffin Sections and Immuno Staining

Rats were anesthetized with sodium pentobarbital and fixed with 4% neutral buffered formalin by cardiac perfusion. The carotid bifurcations were then excised and immersed in the same fixative for 4 hr and embedded in paraffin. Paraffin-embedded sections (3 μm) were cut by a Finesse 325 paraffin slicing machine (Thermo Fisher Scientific, Waltham, MA, United States) and mounted onto positively charged slides.

For immunohistochemistry staining, the sections were deparaffinized by being heated at 45°C for 1 hr, cleared with xylene solution, and then rehydrated with washes in decreasing grades of ethanol (100, 95, 80, and 60%) for 3 min each. After washing with PBS, the slides were incubated in citrate buffer (pH 6.0) for 15 min at 100°C to retrieve the antigen. Then, the sections were immersed in 3% H_2_O_2_ to block endogenous peroxidase activity. After being blocked with 10% goat serum for 1 hr, the sections were incubated overnight at 4°C with the rabbit anti-BACE1 (1:60). After being washed three times in PBS containing 0.2% Triton X-100, the sections were incubated and then visualized with the GTVision^TM^ III Detection System/Mo & Rb Kit (Gene Tech, Shanghai, China) according to the manufacturer’s instruction. A negative staining control was prepared by omitting the primary antibody (data not shown). Then the sections were stained with hematoxylin for 10 s to label the cell nucleus. The sections were then examined with a Nikon H600L microscope and photographed with a Nikon digital camera DS-Fi1c (Nikon, Tokyo, Japan).

For double immunofluorescence staining, the sections prepared as described above were stained with a mixture of two primary antibodies as follows: (1) rabbit anti-BACE1 (1:50) with mouse anti-TH (1:2000); (2) rabbit anti-BACE1 (1:50) with mouse anti-GFAP (1:200); or (3) rabbit anti-BACE1 (1:50) with mouse anti-NF (1:100). After overnight incubation at 4°C, the sections were washed with PBS, and then the slides were incubated at room temperature with a mixture of Alexa Fluro 488 goat anti-rabbit IgG (1:400) and Alexa Fluro 555 goat anti-mouse IgG (1:400). A negative staining control was prepared by omitting the primary antibodies (data not shown). The staining was examined with an Axio Observer A1 microscope and photographed with an AxioCam MRc5 camera (Carl Zeiss, Göttingen, Germany).

### Rat CB Harvest, RNA Extraction and RT-PCR

After being anesthetized by inhalation of isoflurane, the rats were decapitated, and the carotid bifurcations were rapidly removed and placed in 95% O_2_-5% CO_2_ saturated ice-cold PBS. The CBs were dissected from the bifurcation after the surrounding connective tissue was cleaned and then immediately soaked in RNAlater and stored at −80°C until analysis.

Total RNA was extracted from 16 CBs from eight rats using Trizol reagent. After the elimination of genomic DNA, 200 ng of RNA was used for cDNA synthesis by using the QuantiTect Reverse Transcription Kit. The DEPC-H_2_O, instead of RNA, was used in reverse transcription reaction to obtain a negative cDNA control. PCR was completed with the HotStarTaq^®^ Master Mix Kit. Two microliters of cDNA was mixed with 10 μl of HotStarTaq Master Mix and gene-specific primer pairs in a 20 μl reaction volume. The PCR conditions were 95°C for 10 min, followed by 40 cycles at 94°C for 50 s, annealing temperature for 50 s and 72°C for 1 min, followed by 72°C for 10 min. An equal volume of the negative cDNA control was used in the PCR as negative PCR control. The PCR products were loaded onto a 1.2% agarose gel, and then grayscale analyzed by Image J. A total of 3 technical replicates were conducted. The cDNA primers are listed in Table 1.

**TABLE 1:** Sequences of rat primers.

**Gene**	**Accession no.**	**Primer sequence**	**PCR Cycle no.**	**Annealing Tm (°C)**	**Length**
BACE1	NM_019204.2	F: 5′-TCACCAATCAGTCCTTCCGC-3′ R: 5′-GGGCTCGATCAAAGACCACA-3′	35	54.2	174
PGC-la	NM_031347.1	F: 5′-ACTCAGCAAGTCCTCAGTGC-3′ R: 5′-CTCTCTGCGGTATTCGTCCC-3′	35	54.2	277
β-actin	NM_031144	F: 5′-GGGAAATCGTGCGTGACATT-3′ R: 5′-CGGATGTCAACGTCACACTT-3′	35	53	253

*Tm, temperature; F, forward primer; R, reverse primer.*

### Statistical Analysis

The data were presented as means ± S.D. Statistical evaluation was conducted by One-Way ANOVA and Student’s *t-*test. The experimental data were consistent with the homogeneity of variance, and the Bonferroni test was performed as a *post hoc* test to make inter-group pairwise comparison. *P* < 0.05 was considered significant.

## Results

### Expression and Distribution of BACE1 in the CB

**FIGURE 1:**
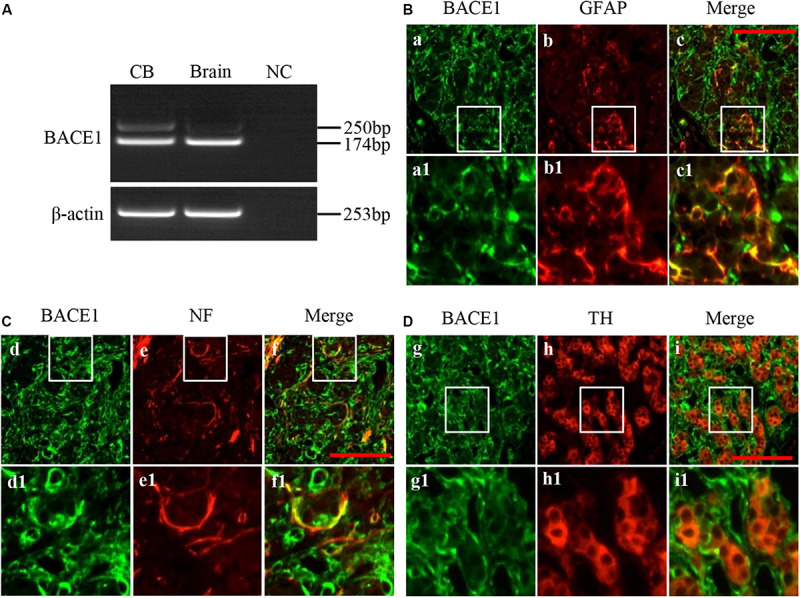
Expression and distribution of BACE1 in rat CB. **(A)** RT-PCR showed expression of BACE1 mRNA in rat CB (left lane). RNA extracted from rat brain cerebral cortex was used as positive control (Brain, middle lane). The DEPC-H_2_O, instead of RNA, was used in RT reaction to obtain a negative cDNA control. An equal volume of the negative cDNA control was used in the PCR as PCR negative control (NC). **(B)** Double immunofluorescent staining of BACE1 (green) and GFAP (red) in rat CB (a-c1). Glial fibrillary acidic protein (GFAP) was used to label CB type II cells. **(C)** Double immunofluorescent staining of BACE1 (green) and NF (red) in rat CB (d-f1). Neurofilament (NF) was used to label CB nerve fibers. **(D)** Double immunofluorescent staining of BACE1 (green) and TH (red) in rat CB (g-i1). Tyrosine hydroxylase (TH) was used to label CB type I cells. a1-i1 are higher magnifications images of rectangle areas in a-i, respectively. Scale bar = 50 μm.

To address whether BACE1 transcript is present in the CB, total RNA from pooled rat CBs was subject to RT-PCR using the primer that would generate DNA fragment with 174 bp. As show in Figure 1A, the expected 174 bp product was detected both in rat CB as well as in the rat brain cortex, which was used as a positive control. The negative cDNA control was used in the PCR as PCR negative control (NC). In the CB, an unexpected product with the length about 250 bp was also observed and the amount of this product is much less than that of the 174 bp product. This result indicates that BACE1 mRNA with different forms probably due to alternative splicing is expressed in the rat CB.

To further characterize the distribution of BACE1 in different cell types within the rat CB, we performed double immunofluorescence staining. BACE1 immunoreactivity was diffusely present in the CB (Figures 1B–D), and colocalized with both GFAP staining (Figure 1B) and NF staining (Figure 1C), whereas BACE1 immunostaining was not observed in TH-positive cells (Figure 1D). These results indicate that BACE1 is distributed in type II cells and nerve endings but not in type I cells in the CB.

### Effect of CIH and ROS on the Protein Level of BACE1 in the CB

**FIGURE 2:**
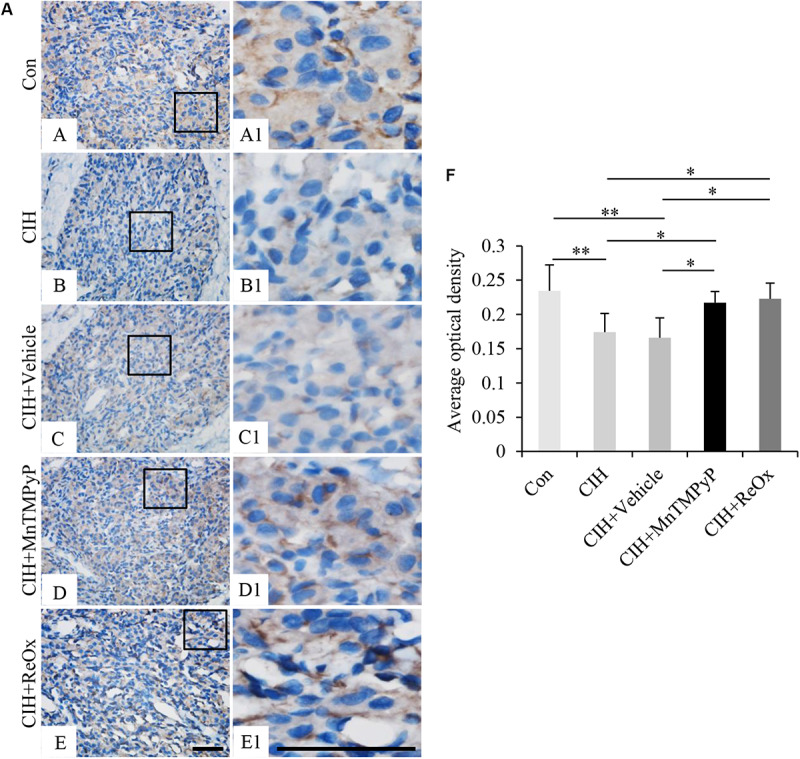
The effect of MnTMPyP and reoxygenation on the BACE1 level in the rat CB. **(A)** Immunohistochemical staining of BACE1 in the CB from Control (Con) **(A,A1**), CIH **(B,B1)**, CIH + Vehicle **(C,C1)**, CIH + MnTMPyP **(D,D1)**, and CIH + ReOx **(E,E1)** group rats. **(A1–E1)** are higher magnification images of rectangle areas in **(A–E)**, respectively. The brown color represents BACE1 staining, while the blue color represents hematoxylin-stained cell nuclei. **(F)** Statistical analysis of BACE1 protein expression levels in rat CB after CIH treatment. The data were presented as means ± S.D. MnTMPyP: scavenger of ROS, CIH: cyclic intermittent hypoxia, ReOx: reoxygenation. *n* = 7 in each group, Scale bar = 50 μm for all images, *P < 0.05, **P < 0.01.

To investigate whether BACE1 expression could be involved in the CIH-induced CB plasticity, by immunohistochemical staining, we observed the expression level of BACE1 in the CB from the rat following a 2 weeks exposure to CIH, and after 2 weeks “recovery” in room air. As shown in Figure 2, CIH exposure for 2 weeks reduced the BACE1 immunoreactive intensity in the CB (Figures 2B,B1, *n* = 7, *P* < 0.01) when compared with that in the control CB (Figures 2A,A1). Moreover, when compared with control, the reduced BACE1 immunoreactive intensity in the CB of CIH rat was restored following return to room air for 2 weeks (Figures 2E,E1, *n* = 7, *P* < 0.05). Because ROS contributes to the consequences of CB plasticity induced by CIH (Peng et al., 2003), we employed MnTMPyP, a superoxide anion scavenger, to assess the potential role of ROS in the inhibitory effect of CIH on BACE1 expression in the CB. The CIH-induced reduction of BACE1 immunoreactive intensity (Figures 2D,D1) was observably returned to control level in the CB from 2 weeks CIH rat with daily treatment with MnTMPyP (*n* = 7, *P* < 0.05). These results reveal that CIH reversibly reduces the protein level of BACE1 in the CB and this effect might be mediated by ROS.

### Effect of CIH on mRNA Expression Levels of BACE1 and PGC-1α in the CB

**FIGURE 3:**
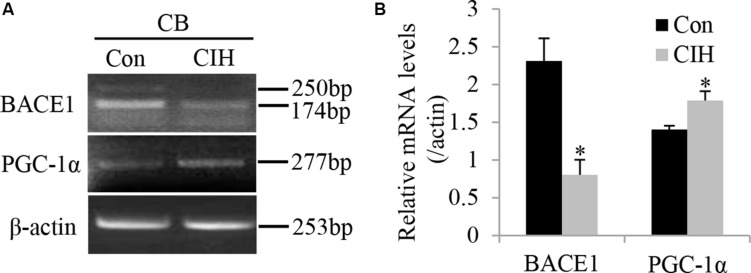
CIH treatment decreases the BACE1 mRNA level and increases the PGC-1α mRNA level. **(A)** RT-PCR showed expression of mRNAs of BACE1 and PGC-1α in rat CB taken from Control (Con, left lane) and CIH (right lane) group rats. **(B)** Statistical analysis of BACE1 and PGC-1α mRNA expression in rat CB after CIH treatment. The data were presented as means ± S.D. CIH: cyclic intermittent hypoxia. *n* = 8 in each group, *P < 0.05.

The major adaptive response to hypoxia is the transcriptional activation of various genes, which includes transcription factors, such as PGC-1α. Katsouri et al. (2011) previously reported that PGC-1α decreased BACE1 expression and transcription by inhibiting BACE1 promoter activity. To determine the effect of CIH on the mRNA expression level of BACE1 and PGC-1α, we conducted RT-PCR to examine the mRNA expression level of BACE1 and PGC-1α in the CB after CIH exposure for 2 weeks. Figure 3A shows that CIH reduced the mRNA level of BACE1 (*n* = 8, *P* < 0.01) but augmented the mRNA level of PGC-1α (*n* = 8, *P* < 0.05).

## Discussion

Here, we found for the first time that BACE1 was expressed in the CB and distributed in the nerve endings and type II cells, but not in the type I cells. CIH reduced BACE1 level in the rat CB, and reoxygenation or a ROS scavenger alleviated this reduction. Moreover, we found that CIH augmented the mRNA level of PGC-1α but attenuated the mRNA level of BACE1 in the CB. Considering the known function of BACE1 and the PGC-1α-dependent transcriptional inhibition of BACE1, we speculate that CIH promotes the production of ROS to upregulate the level of PGC-1α, which in turn inhibits the transcription of BACE1, and that a reduction in the BACE1 level may be related to CIH-induced CB plasticity.

The CB, as a major peripheral chemoreceptor, detects changes in oxygen levels in arterial blood. CIH has been demonstrated to lead to serious pathophysiological consequences (Nieto et al., 2000; Peppard et al., 2000; Prabhakar et al., 2005). Peng et al. (2003) found that exposure to CIH for 10 days evokes the sLTF of CB sensory activity in response to acute intermittent hypoxia. This plasticity is CIH duration-dependent and a completely reversible phenomenon. It has been demonstrated that ROS generated by NADPH oxidase is required for CIH-evoked plasticity of the CB (Peng et al., 2009). However, the downstream signal molecules of ROS in this process are undefined yet.

BACE1 has been widely studied in the pathogenesis of AD. As an aspartyl protease, BACE1 cleaves amyloid precursor protein (APP) into amyloid-beta (Aβ), the main component in amyloid plaques. Accumulating evidence indicates that BACE1 is also involved with synaptic plasticity by influencing the formation of peripheral nerve myelin, debris clearance, and axonal regeneration (Willem et al., 2006; Farah et al., 2011; Tallon and Farah, 2017). BACE1 gene is composed of nine exons and its pre-mRNA was found to undergo complex alternative splicing (Mowrer and Wolfe, 2008; Fisette et al., 2012). The full-length of 501-amino acid protein (BACE1 501) is the major isoform of BACE1 protein. In the brain, although it has been thought that BACE1 is highly expressed in neurons, BACE1 expression is also observed in reactive astrocyte (Rossner et al., 2005). In this study, we demonstrate that the mRNA of BACE1 is expressed in the peripheral chemoreceptor CB. We have found that there are two PCR product binds of BACE1 in the CB (Figure 1A), indicating that a splicing variant of BACE1 probably exist in the CB. Further characterization of alternative BACE1 splicing in the CB is needed. Here, we showed that BACE1 was mainly distributed in the GFAP-positive type II cells as well as the NF-positive nerve endings, but not in TH-positive type I cells (Figures 1B,C). BACE1 is a transmembrane protease and the localization of its substrate proteins are on the plasma membrane of its own cells as well as adjacent cells. Thus, we speculate that the distribution of BACE1 in the CB is more beneficial for BACE1 to participate in the regulation of CB synaptic plasticity after CIH.

Several validated substrates of BACE1 have been demonstrated to be involved in peripheral nerve synaptic function. For instance, BACE1 promotes peripheral nerve myelination and axonal bundling by processing type III NRG1 or CHL1 and L1. *BACE1*^–/–^ mice display hypomyelination of peripheral nerves and synaptic dysfunction (Willem et al., 2006; Hitt et al., 2012; Filser et al., 2015; Munro et al., 2016). Interestingly, some evidence has shown that a reduction in BACE1 activity is also involved in the promotion of axonal regeneration in injured peripheral nerves. For example, Tallon and Farah found that decreased level of BACE1 downregulates the cleavage of CHL1 and L1 so that more full-length CHL1 and L1 are distributed on the cell surface to interact with Schwann cells, enhancing the regeneration of the injured peripheral nerve (Tallon and Farah, 2017). Thus, BACE1 acts like a double-edged sword in the peripheral nervous system and has two-way effects. In this study, we found that CIH attenuated the expression level of the BACE1 mRNA and protein in the CB (Figures 2, 3). The decrease in BACE1 level might reduce the cleavage of its substrate such as APP to reduce the neurotoxicity after CIH treatment. Additionally, the decrease in BACE1 expression may also accelerate the clearance of debris and promote axonal regeneration in ROS-induced injured sensory nerve endings as a compensatory mechanism. These may be beneficial to the formation and maintenance of CB plasticity induced by CIH.

In this study, one cycle of CIH, modeling the gas exchange abnormalities of OSA took 6 min, so the number of CIH episodes was 10 times per hour. According to the average number of sleep apnea-hypopnea index (AHI) per hour, OSA is divided into mild (AHI, 5–15/hr), moderate (15–30/hr), and severe (>30/hr) paradigm. Therefore, CIH paradigm in this study is compatible with mild clinical OSA. It is known that the outcomes of OSA depend on AHI severity. In CIH animal model, the effect of CIH is generally related to the hypoxia severity, CIH episodes per hour as well as the duration of CIH (Li et al., 2007; Lim et al., 2016). Peng and colleagues (Peng et al., 2003) have reported that CIH (9 episodes per hour, 8 hr/day) evoked sLTF of carotid body in rats with ROS-involved and time-dependent reversible manner. However, they found that varying the FiO_2_ from 10 to 5% with same CIH episodes and duration did not impact the strength of sLTF of carotid body. Here, we found that CIH-induced reduction of BACE1 level is also a ROS-involved reversible phenomenon. Taken together, we speculate that the effect of CIH on BACE1 level may not be related to the extent of hypoxia within a certain range of hypoxia, but related to the duration of CIH cycle which may result in ROS production with diverse levels. It has been demonstrated that the roles of ROS in signaling is related with its cellular abundance (Kalogeris et al., 2014). A mild increase in ROS may be beneficial to the maintenance of homeostasis *in vivo*, while ROS level under severe CIH may injury tissue cells. Short-term CIH with mild ROS level induces the reversible BACE1 reduction, while long-term CIH with severe ROS level may lead to persistent reduction of BACE1, or increase BACE1 level by activating other signal pathways. Several BACE1 substrates have been validated having opposite effects on the peripheral nerve (Willem et al., 2006; Hitt et al., 2012; Filser et al., 2015; Munro et al., 2016; Tallon and Farah, 2017), indicating that BACE1 has two-way effects in the peripheral nervous system. Thus, we speculate that the reversible decrease of BACE1 level probably contributes to the maintenance of CB sLTF; while, the persistent reduction of BACE1 level or the increase of BACE1 level may impair the CB function. It is must be noted, however, the effects of long-term CIH paradigm or different hypoxia severity of CIH on the level of BACE1 in the CB, and the effect of BACE1 on the CB are all needed to be further clarified.

The regulation of BACE1 is affected by many factors. H_2_O_2_-mediated oxidation induces BACE1 expression via transcriptional activation and results in increased enzymatic activity (Kwak et al., 2011). PGC-1α inhibits the expression of BACE1 by repressing transcription in the BACE1 promoter region (Wang et al., 2013), or by enhancing BACE1 ubiquitination and proteasome degradation (Gong et al., 2013). Although, it has been demonstrated that PGC-1α initiates an anti-ROS program to prevent intracellular ROS production under oxidative stress condition, PGC-1α is also induced by treatment with oxidative stressor (St-Pierre et al., 2006). It has been reported that ROS upregulates mRNA level of PGC-1α by activating AMPK in skeletal muscle cells (Irrcher et al., 2009). In this study, we found that CIH increased PGC-1α mRNA expression (Figure 3). Thus, we speculate that CIH facilitates ROS production, which in turn causes a compensatory enhancement of the multifaceted ROS defense system, including the activation of PGC-1α transcription. It has been reported that PGC-1α inhibits BACE1 transcription by binding to the -1357/-1333 PPRE site in the negative regulatory region of BACE1 promoter (Wang et al., 2013). In our study, CIH decreases BACE1 mRNA expression (Figure 3). Based on these results, we speculate that CIH inhibits BACE1 mRNA expression probably via ROS-induced activation of PGC-1α transcription. It is important to note that our data from Figure 3 does not support PGC-1α directly regulating BACE1 transcription. Using PGC-1α knockout transgenic mice may provide an approach to clarify the direct effect of PGC-1α on CIH-induced downregulation of BACE1 mRNA. Accumulating evidence demonstrate that BACE1 promoter contains numerous functional transcription factor binding sites (Sun et al., 2005), such as SP1, YY1, and AP1. Thus, BACE1 transcription is affected by multiple transcription factors. For example, SP1 activates BACE1 transcription by binding to its specific sequence at -911/-906 region in BACE1 promoter (Christensen et al., 2004.) Interestingly, it has been reported that ROS inhibits SP1 transcription in a p53/miR-335-5p-dependent manner (Lu et al., 2020). Therefore, ROS may also contribute to the CIH-reduced mRNA expression of BACE1 in the carotid body through inhibition of SP1 transcription. Similarly, the direct evidence of ROS regulating PGC-1α transcription in the carotid body is also needed to be provided. In our next study, we will use ROS scavenger MnTMPyP to observe whether CIH regulates PGC-1α mRNA and protein levels by facilitating ROS production. On the basis of previous studies and our findings, additional studies characterizing the mechanism underlying the effect of CIH on BACE1 transcription in the carotid body is needed.

## Conclusion

In conclusion, we have identified BACE1 in the CB and correlation of BACE1 level in the CB with generation of ROS in the CIH-exposed rat. These data provide a possible peripheral modulatory role of BACE1 on CIH-evoked CB plasticity, which must be further addressed in the future.

## Data Availability Statement

The raw data supporting the conclusions of this article will be made available by the authors, without undue reservation, to any qualified researcher.

## Ethics Statement

The animal study was reviewed and approved by the Institutional Animal Care and Use Committee of The First Affiliated Hospital of Xinxiang Medical University.

## Author Contributions

CL, BZ, and YL contributed to the conception and design, data analysis, and manuscript writing. CL, BZ, YF, and XJ contributed to the experiments performing. CL, BZ, YF, and YL contributed to the collection and assembly of data. CL, BZ, YF, XJ, and YL contributed to the final approval of manuscript. All authors have read and agreed with the manuscript.

## Conflict of Interest

The authors declare that the research was conducted in the absence of any commercial or financial relationships that could be construed as a potential conflict of interest.
